# Editorial: Editors’ showcase 2021/2022: insights in cell adhesion and migration

**DOI:** 10.3389/fcell.2023.1216701

**Published:** 2023-06-12

**Authors:** Claudia Tanja Mierke

**Affiliations:** Faculty of Physics and Earth Science, Peter Debye Institute of Soft Matter Physics, Biological Physics Division, Leipzig University, Leipzig, Germany

**Keywords:** migration, adhesion, mechanical properties, N-cadherin, metatonin, Alzheimer’s disease, neuroregeneration

The Editor’s Showcase 2021/2022 is not a typical Frontiers in Cell and Developmental Biology Research Topic in Cell Adhesion and Migration. It emphasizes exceptional manuscripts that are of value to a diverse group of researchers and have been written or co-written by our editors. The Research Topic is not assigned to any subject area and therefore covers different areas. This special Research Topic of the Cell Adhesion and Migration section of the Journal Frontiers in Cell and Developmental Biology comprises a total of five articles, including one original article, brief research report and three reviews.

The original research article by Nagasaka and Miyata investigates the impact of mechanical cues on the development of the brain wall by atomic force microscopy (AFM). The inner/apical surface of the embryonic brain wall represents an essential niche for the generation of neural progenitor cells (NPCs). Nagasaka and Miyata reveal that the elastic modulus at a concave-shaped apical surface of the pallium is higher than at a convex-shaped apical surface of the ganglionic eminence (GE). Based on AFM measurements, it has turned out that the contribution of actomyosin, as evidenced by the softening of the apical surface by blebbistatin and the stiffness of dissociated individual NPCs, is comparable between pallium and GE. So, the difference in the stiffness of the apical surface, which is higher in pallium than in GE, cannot be accounted for by differences in the contribution of the interaction between actin and myosin. Subsequently, they determine that the density of apices of NPCs is increased in the apically stiffer pallium and exhibits a denser F-actin reticulation than in GE. A plausible explanation for the difference in stiffness between palladium and GE at the apical surface could be due to a difference in densification of the NPC apices. During laser ablation of the apical surface, the convex apical surface of GE exhibits more accelerated edge retraction than the apical surface of pallium, with less retraction restrained by blebbistatin than in pallium. This stronger pre-stress in GE may suggest how the originally apically concave wall subsequently turns into an apically convex entity.

In a brief research report, Hagiyama et al. describe the potential therapeutic usage of anti-Cell Adhesion Molecule 1 antibodies in the treatment of the malignant and highly aggressive pleural mesothelioma. Malignant pleural mesothelioma (MPM) cells, such as NCI-H28, NCI-H2052, MSTO-211H, Meso-1-CADM1, MESO-4, MESO-9, MESO-14, and EHMES10, express detectable protein amounts of the cell adhesion molecule 1 (CADM1), while no expression could be detected for four other cell lines. Using immunofluorescence imaging the expression of CADM1 can be seen on the plasma membrane surface of cells, such as MCI-H2052 and MESO-14, within a monolayer. Mimicking *in vivo* growth of MPM plaques on the pleural surface, a coculture of MPM cells on mesothelioma cells have been performed. Evidence emerged that MESO-1-CADM1 cells proliferate more than the original MESO-1 cells, indicating that CADM1-mediated cell adhesion is implicated in the growth of MPM cells on top of mesothelial cells. The addition of neutralizing anti-CADM1 antibodies (9D2) to these two cell lines growing on a confluently single layer of mesothelial MeT-5A cells (express the trans-binding partner CADM4, but not CADM1) results in decreased growth. Similar to antibody-drug conjugates (ADCs) for nectin-4, which is highly similar to CADM1, that promote aggregation and internalization, a CADM1 ADC (h3E1-MMAE) enhances the growth suppressive activity by inducing aggregation and internalization of CADM1.

In a review article on the role of the hormone melatonin in Schwann cell plasticity and as a possible causative agent of circadian rhythmicity in neuroregeneration, Klymenko and Lutz discuss current hypotheses and concepts. Particular attention is given to the effects of Schwann cell plasticity and its controlled phenotype sequence on neuroregeneration. Specifically, whether melatonin modulates Schwann cell activity during the course of neurorepair and whether circadian control and rhythmicity of Schwann cell function plays a key role in neuroregeneration. The importance of the two melatonin receptors on Schwann cells is also discussed. Either an intrinsic functional rhythm or a systemic circadian clock may provide precise timing and accuracy of signaling pathways in the nervous system regeneration process. Circadian rhythmicity is discussed as a potential contributor to neurorepair. In addition, it is indicated that melatonin alters the extracellular matrix (ECM) scaffold, such as collagen, which has been found to be crucial for the myelination processes of neurons. In essence, the microenvironment of Schwann cells is vital to their functioning role. For example, injury of the nervous system can alter the expression and activity of matrix-metalloproteinases (MMPs) that impact the motility of Schwann cells. Finally, the authors concluded that melatonin can trigger dramatic alterations within Schwann cells, among them are induction of dedifferentiation, locomotion, and impairment of glial scar creation.

In the unique and clearly focused review paper by Blaschuk, the therapeutic use of antagonists and agonists of the cell-cell adhesion molecule N-cadherin is discussed. The review article describes the role of N-cadherin in cell-cell adhesion and signal transduction events. The N-cadherin interactome is characterized. In addition, the effect of N-cadherin clustering is presented and how it is regulated. Emphasis is placed on the application of these compounds, namely, N-cadherin antagonists (inhibitors), in cancer treatment, fibrosis, and regulation of vascular function, such as tube formation in Matrigel. Thereby, the results of antagonists and agonists are summarized in animal models. Biomaterials, such as the ECM of the cancer stroma, containing these N-cadherin antagonists (e.g., compound 15) are also examined in terms of their function in remodeling fibrotic tumors, inducing interference between cancer cells and T cells, and delivering drugs to solid tumors. In addition, the effects of the N-cadherin antagonist on endothelium-endothelium and endothelium-pericyte interactions are presented.

The extraordinary and complex review by Pfundstein et al. deals with the interaction of amyloid precursor protein (APP) and amyloid- β (A β ) with cell adhesion molecules under normal physiological conditions and Alzheimer’s disease. Alzheimer’s disease is known to be an untreatable neurodegenerative disorder in which malfunction and depletion of synapses and neurons result in cognitive deterioration and death. The buildup and aggregation of neurotoxic Aβ-peptides produced through amyloidogenic processing of APP have been found to take a pivotal part in the cause of the disease. APP interferes with cell adhesion molecules that affects the normal physiological features of APP, its amyloidogenic and nonamyloidogenic downstream processing, and the production of Aβ-aggregates. The cell adhesion glycoproteins molecules at the surface mediate also Aβ binding to the neuronal cell surface and trigger intracellular cues that participate in Aβ toxicity. In this review, current evidence on the interactions of cell adhesion molecules with APP and Aβ is discussed and the supporting evidence for their pivotal in governing APP processing and physiological functionality, as well as Aβ toxicity, is parsed. Several cell adhesion receptors, such as integrins, interact with APP. In addition, β1-integrins, E-cadherin, N-cadherin, and neurexin have a function in processing APP. The authors pointed out that it is still necessary to comprehend the normal functions of APP, and regulation of its processing, to uncover the mechanisms behind Aβ-induced toxicity. Otherwise, treatment options for Alzheimer’s disease will not improve. Finally, the authors concluded that this is a necessary part of the complex Alzheimer’s scenario that should be grasped to design safe and effective therapeutic treatments for Alzheimer’s disease.

These five articles demonstrate the complexity of the various approaches and subfields in cell adhesion and migration research. The biochemical and mechanical characteristics of cells, and tissues have been explored using very different methods.

There are many other interesting Research Topics to explore, such as whether the microenvironment has a unique effect on cellular functions and signaling pathways. The wide variety of techniques and methods used in the different investigations suggests that understanding the microenvironment and its mechanical cues is urgently necessary to achieve a comprehensive picture of cell adhesion and migration.

